# Cross-cultural science: ten lessons

**DOI:** 10.3389/fmicb.2015.00681

**Published:** 2015-07-14

**Authors:** Joanne M. Horn

**Affiliations:** Global Bioengagement, MRIGlobal, Frederick, MD, USA

**Keywords:** bioengagement, partnering, capacity building, biosurveillance, global health security, disease outbreak prevention

## Abstract

Concerns of infectious disease outbreaks have recently reached the forefront of global security issues and resulted in new engagements among foreign science advisors, host country scientists, and officials. There are lessons to be learned from the numerous organizations working in global regions of endemic disease who are building capacity to survey pathogens and prevent and contain epidemics. Working with foreign scientists, health professionals, and administrators can be challenging; building partnerships based on respect and mutual trust is key to achieve effective change. Engendering ownership, working toward mutual success, paying close attention to cultural norms and the local regulatory climate, close collaboration with other stakeholders, and imaginative problem solving all contribute to mission success.

## Introduction and Background

In recent years, many factors have converged that have collectively increased the threats of pandemic infectious disease. The globalization of trade and travel, expanding use of wildlife resources and habitats by humans, cultural practices bringing livestock and people into close contact, episodes of war and terrorism, and climatic change have focused our collective awareness of the continuing need for engagement to strengthen global health security. A number of national and international programs are operating throughout the world with mandates to sustainably improve biosafety practices, disease surveillance capacity, outbreak response and reporting, and supporting and sharing research activities. These include such organizations as Global Health Partnership; the European Union Chemical, Biological, Radiological, and Nuclear Centers of Excellence Initiative; the United Nations-sponsored groups, World Health Organization, the World Organization for Animal Health, and Food and Agriculture Organization; and U.S. Government agencies spanning the Departments of Defense, State, Health, and the U.S. Agency for International Development. Other organizations such as the President’s Emergency Plan for AIDS Relief (PEPFAR) and the Global Fund to Fight AIDS, Tuberculosis, and Malaria have been instrumental in targeting specific infectious disease that affect a broad swath of humanity in the developing world. The Global Health Initiative has sought to strengthen public health systems worldwide. In pursuit of these goals, global health professionals have fanned out across the globe, establishing relationships with local infectious disease scientists, clinicians, research institutes, public health laboratories, hospitals, and government officials in focus countries and regions.

Many of these efforts have been guided by the evolution of the goals of global health policy since the end of the Cold War, from those seeking to advance political and economic advantage, to seeking security and stability (Barnes and Brown, 2011). Driven by deliberative-based theories of inclusiveness for driving collective decisions (Brown, 2010), and practical awareness of effective methods to achieve development goals, the principle of establishing true partnerships with aid recipients was actively promoted in the mid-1990s (Barnes and Brown, 2011). The following decade brought the concepts of pursuing target country ownership of health programs, along with a more or less defined path toward capacity building: to assess, plan, monitor, and evaluate (Goldberg and Bryant, 2012). Along the way, many challenges and requirements have been documented to make these principles work, including jointly defining goals and metrics, the transference of operations to local control mechanisms, and the need to integrate the efforts of all aspects of public health infrastructure with disease detection (Garrett, 2007).

MRIGlobal, as a (not-for-profit/independent research) contractor, has been providing support for the Defense Threat Reduction Agency’s Cooperative Bioengagement Program for the last 10 years, and also helped support global health security missions in the Middle East, South Asia, and Africa over the last 20 years. Executing these science-based programs has put us into direct continuing contact with local collaborators in infectious disease science, and consistently reinforced the absolute requirement to establish collegial relationships with our foreign partners to successfully execute programmatic objectives. Along the way, we have learned many lessons—some hard won—in communication and understanding across the cultural divide, and gained important insights from the many individuals engaged in these programs. We share here some of what we and others have discovered as an aid in the ongoing development of effective mechanisms for the near term, and to ultimately reduce the infectious disease burden globally.

Our experience is certainly not unique. Many other accomplished professionals have applied serious analysis and relayed formulations to successfully improve the capacity for detecting, responding and preventing infectious disease (or other risks, such as nuclear threats) throughout the developing world. These particular guidelines were chosen because they are fundamental, and so generally apply to human relationships across the board, in business, policy, or technology; but they seem especially essential for successfully changing views and behaviors across vastly different cultures. The reward of success in these programs, as Harold Varmus states, “is a connection made to the culture of science, in which decisions are based on evidence…and interactions among people of varied backgrounds are grounded in common goals” to improve global health security (Varmus, 2014). Investing in these relationships, in short, can help improve the health and well-being of countless lives, and bolster our collective security from disease risks.

## Guides to Constructive Partnering

(1) (1) **Mutual respect is foundational.** While corporate and academic environments in the U.S. may lend themselves to productive adversarial or competitive approaches as key to success, these are counterproductive in establishing working relationships in most foreign cultures. Approaching potential partners with an appreciation and open respect of their knowledge and skills builds mutual understanding and trust. As has been noted, global health professionals need to value individual and organizational representative capacity, as well as technical competence (Kevany, 2014), and function as capable health diplomats to be successful (Novotny and Adams, 2007). A partners’ willingness to cooperate, listen, and follow through with meaningful action is ultimately rooted in shared trust. No matter how profound the individual differences may seem, a personal connection is valuable when seeking mutual areas of interest and investment, to allow all parties to reach a position of shared purpose.

One indicator of achieving trust is a continued partnership, which can offer reward. A scientist who engaged a national Laboratory in West Africa and helped solve their cold-chain supply issues by facilitating installation of a nitrogen generator, was later called back to respond to the Ebola crisis and provided an “incredible amount of data” that wasn’t accessible previously (Rozo, 2014). Trusting partnerships can provide low-key cooperative relationships even while government-to-government negotiations are in flux, and offer reality checks on what truly constitutes a security risk (Franz and Lehman, 2009).

(2) **Ownership is essential.** “Country Ownership” is a capacity building strategy that shifts the leadership and responsibility for enacting systematic improvements onto the partner country and includes local involvement (Goldberg and Bryant, 2012). The most effective way to affect change is for a partner to adopt your goals and objectives as their own. They will ideally realize that outcomes will enable advances for individual and societal benefits—whether social, personal, political, economic, or strategic. Partners should take possession of the cause and advance it as their own. This “personalization of objectives” is a critical step toward sustainable programs. Without ownership, the associated programs and objectives will be abandoned once the foreign sponsor ceases involvement. Partner investment and ownership is essential to embed programs into national science and governance, and true partnerships are requisite to conduct systematic assessments, analyze challenges, define goals, prioritize action, and implement activities (Goldberg and Bryant, 2012).

In June, 2012 a group of U.S. science and engineering students and an experienced group of researchers from Yemen came together in Jordan under the auspices of the Federation of American Scientists’ International Science Partnership. As they worked through issues to address Yemen’s energy and water dilemmas it became clear that the Yemeni scientists were not only interested I furthering humanitarian concerns, but also “exploring new ideas, learning from peers, and gaining experience to further their own careers” (Jansson and Ferguson, 2012). While this is not surprising, it does demonstrate that ownership can be achieved from a number of directions.

(3) **Remain perceptive.** While the term “cultural sensitivity” has become a cliché, it is vital to appreciate why people speak or act as they do. Listen actively and cultivate an awareness of nonverbal cues such as body language; and consult with sympathetic local nationals to get an idea of whether cultural customs may be playing a part in your interactions (Katz et al., 2014). By a common example, it is considered polite to exchange pleasantries and inter-personal banter prior to starting a meeting. Despite our Western urges to get on with the agenda and limit our introductions to 30-s elevator speeches, this approach is widely considered rude by others. Regarding hierarchical systems, an individual’s professional background determines the credence given to all later statements in a meeting. Also, due respect must be paid to senior members of any delegation. Partners can be protégés of their respective patrons throughout their careers—they may have little individual power to make decisions without the full agreements of their boss. Overall, developing a knowledge of foreign cultures, values, and norms is key to success (Katz et al., 2014) and adaptation to local needs and customs is often essential (Kevany et al., 2012).
(4) **All the players matter.** Efforts to build and sustain programs depend not only on the immediate relationships you build with local individuals but also with governmental representatives, non-government organizations, international organizations, other agencies, and contractors (Atun and Kazatchkine, 2009). There is often much to learn from fellow stakeholders, and cooperation helps avoid wasteful duplication of effort while working toward mutual goals; multilateral coordination is very helpful (Katz et al., 2014). Other organizations—especially well-established ones—can frequently help navigate the terrain if mutual wins are sought. Despite these efforts, the interests of individuals, organizations and governments may fail to align and the net benefits may result in their unequal distribution creating both winners and losers (Smith, 2014). In some situations, local institutions may try to disrupt or prevent communication between different stakeholders. Only frequent, independent, often confidential, exchanges between stakeholders can prevent double-dipping by recipients and allow stakeholders to best leverage collective resources and find synergies.
(5) **Ethical conduct is key.** Do not promise what you cannot deliver; if you need to consult the sponsor, upper management, or other stakeholders, then disclose that fact; be transparent. There are many instances where there is misunderstanding of the uncertainty of the flow of knowledge globally (Anderson, 2002), which might be avoided by a clear explanation of how, why, and where knowledge is disseminated. Scrupulous attention to fairness pays; often, local institutions are competing for scarce resources. Limit your discussion to the institution you are dealing directly with to avoid political infighting. On the institutional level imparting the “3 Cs of Biosecurity: Codes of Ethics, Codes of Conduct, and Codes of Practice” (Nasim et al., 2013) starts with the relationship between individuals; be an example of what you hope to create.
(6) **Work-arounds to rejection.** Confronting a wall of refusal, denial, deception, or other misdeeds is frustrating and can be threatening to mission success. While the urge may be to directly confront the individuals delivering the message, active listening to understand the underlying issues may be more productive (Katz et al., 2014). There may be alternative explanations or motivations that lend themselves to resolution. For example, management or policy forces your partner to react, local law prevents action, or traditional corruption or patronage pathways are obstructive. Often, approaching the immediate partner face-to-face and asking what might be acceptable to their management, along with offering alternatives, is a successful path to conflict resolution. For example, we were once confronted with refusal to export microbial strains from a country we engaged with, preventing any progress on an entire joint research program. We worked out alternatives—raw sample transfer, DNA transfer, data transfer—and asked our local project collaborators what they thought their ministries would accept. Our sponsors took that information to the ministries, and we were able to negotiate a compromise. In summary: Stay flexible and develop alternate plans for reaching your goals.

As a corollary, be careful if you are considering citing someone’s support for a concept directly to their management. In other words, do not argue to upper levels of management that they should buy into an idea because their underlings agree. This approach may fly I the face of hierarchal decision-making and backfire, as well as put allies at risk.

In a time when pathogen discovery and disease disclosure can have serious political, diplomatic, economic, or military implications, extreme caution needs to be exercised in approaching the right authorities with the correct information, and offer constructive, accurate, and actionable solutions. The emergence of influenza strain H5N1 in Indonesia, for example, caused the sequestration of critical viral samples (“viral sovereignty,” among other downstream events, Elbe, 2010; Smith, 2014); such reactions can potentially have disastrous consequences.

At the end of the day, health diplomats whether clinical, scientific or policy-makers, need an understanding of the structures, programs, approaches, and pitfalls of their relationships (Novotny and Adams, 2007). As one illustration, the International Science and Technology Center (ISTC), an multi-country consortium that seeks to counter proliferation, has had their projects curtailed in Russia due to political mistrust. This situation may be compounded by the demise of influence of scientists and their influence on policy (Bergstrom, 2011).

(7) **Don’t rely on individuals.** Political and management structures can rapidly change, and while you take time to build and cultivate good interpersonal relationships, trust is not necessarily a transferable commodity (Smith, 2014). Expect to educate new players as they come onboard; this may also translate to educating their entire staff. Patronage systems often involve wholesale change: When one chief is dismissed, all the other subordinates can fall, too. It has also been pointed out that whatever legitimacy is forged among scientists must be transferred to policy setters to have a larger effect, and that transmission requires connectivity; trust can be difficult to transfer (Smith, 2014). Harold Varmus has in fact proposed that while admirable efforts to build capacity in Uganda and Mali, these have been overtaken at times by larger political forces (Varmus, 2014).
(8) **Know the regulatory context.** Understand limitations of local and national statute; you will need to work within these to keep the program from running aground and your partners cooperative. The Center for Science, Technology, and Security Policy (AAAS) implemented a cooperative research grant program in the broader Middle East and North Africa in 2010, and specifically pointed out that areas of concern may range from ethical research practices, to export control, contract law, and transport regulations (Coat et al., 2013). These issues become magnified when regional projects involving more than one country are encountered. The formulation of new laws may be possible if the right people and institutions can be involved; or if it is possible to incorporate important revisions of current laws by merging international norms with current local law.
(9) **Pay Attention to metrics.** Programmatic metrics and evaluations of effectiveness are important when gaging success —it is equally critical to impart these skills and approaches to target partners. Selecting key indicators of success and unbiased self-evaluation will help to continually improve programs, even after the sponsor is gone. As with other aspects of engagement, flexibility is key. If the engaged partners are not willing to share a key piece of information out of security or other concerns, try to get agreement on an alternate parameter that is acceptable. It has been reasonably argued that a body of evidence –based metrics should be built around what works to build disease surveillance and outbreak response (Goldberg and Bryant, 2012). Identify indicators that measure change over time (Honadle, 1981) and are truly representative and reflect the goals of the program as identified in the initial assessment phase and promote targeting areas that require further investment. Indicators can vary and include process, output and impact indicators (Goldberg and Bryant, 2012). While it can be difficult to identify appropriate indicators for “soft” program results, such as improving prioritization of risk, these are critical to assess (Franz and Lehman, 2009).
(10) **Outliers can be central.** Institutions or individuals who are not primary programmatic or contractual targets may end up being fundamental to achieving sustainability. If you approach these players, there can be big payoffs. Never turn away a potential participant without carefully considering what they can bring to bear. If you approach them independently, they may well be honored—and there may be benefits to them and their institutions. Such is often the case, for example, with universities educating the pipeline of new leaders. Makerere University’s Ugandan Cancer Institute survived through the Amin dictatorship largely due to the heroic efforts of Charles Olweny, who was mentored early in his career by colleagues at the U.S. National Cancer Institute and Sweden’s Karolinska Institute (Varmus, 2014). Strive for a broad but effective reach within the limitations of schedule and budget, and with sponsor approval.

Engaging with foreign cultures to generate cultural scientific change and promote genuine improvements in human and animal health can be both frustrating and rewarding. Continuous recycling back to the same issues, dealing with a revolving door of officials, stonewalling, and sometimes plain abject rejection are all challenging. However, accept that change will be incremental and that good ideas often take years to have an impact, even in our own systems. Some estimate that it will take at least a full generation or more to substantially improve public health in some of the developing world (Garrett, 2007). Maintain patience; keep educating as new players enter the scene. Stay positive; soured relationships rarely turn around once mistrust is established. If you have the opportunity to work long-term on a program, there is satisfaction in the long view back, where friendships have been forged, colleagues gained, and the path toward change is being realized.

### Conflict of Interest Statement

The author declares that the research was conducted in the absence of any commercial or financial relationships that could be construed as a potential conflict of interest.
